# Designing Effective Protocol-Based Pharmacotherapy Management: Assessment of the Development Processes and Outcomes in Inflammatory Bowel Disease Care Prescription Management

**DOI:** 10.3390/pharmacy13010017

**Published:** 2025-02-04

**Authors:** Masatsugu Sato, Shiho Fujita, Masahiko Kimura, Ken Takeuchi, Yukihiro Hamahata, Yoshikazu Matsuda

**Affiliations:** 1 Division of Clinical Pharmacology, Graduate School of Pharmaceutical Science, Nihon Pharmaceutical University, Ina 362-0806, Japan; msato@gpro.com (M.S.); m-kimura@jichi.ac.jp (M.K.); 2 Pharmacy Department, Tsujinaka Hospital Kashiwanoha, Kashiwa 277-0871, Japan; fujita@gpro.com (S.F.); ken.takeuchi@gpro.com (K.T.); hamahata@aol.com (Y.H.)

**Keywords:** inflammatory bowel disease, protocol-based pharmacotherapy management, pharmacist-led prescription

## Abstract

Prolonged working hours among physicians in Japan, alongside rising inflammatory bowel disease (IBD) cases, have heightened the need for additional support in IBD care. Protocol-based pharmacotherapy management (PBPM) has emerged as an effective approach that allows pharmacists to assist in prescription management under predefined protocols, potentially reducing physicians’ workload. However, the detailed process of formulating PBPMs remains unclear. This study developed effective PBPM protocols by reviewing past provisional prescriptions. Provisional prescriptions made by pharmacists based on verbal instructions from physicians were reviewed to develop new PBPMs at Tsujinaka Hospital, Kashiwanoha. We retrospectively analyzed the PBPM application rate during three months before and after this initiative based on the proportion of prescriptions processed under standard procedure (SP), pharmacist provisional prescribing (PPP), and PBPM (PBPM-P). A total of 1259 prescriptions were retrospectively analyzed in this study. Before the initiative, there were 586 prescriptions (oral/topical, 128; injection, 458); after the initiative, there were 673 prescriptions (oral/topical, 242; injection, 431). The pre-initiative rates for SP, PPP, and PBPM-P were 68.3%, 30.7%, and 1.0%, respectively. Post-initiative, the rates were 48.3%, 26.6%, and 25.1%, respectively. A significant decrease was observed in the proportion of SP and PPP, while PBPM-P showed a significant increase after the initiative. Specifically, the proportion of PBPM-P increased by 24.1 percentage points, reflecting its broader adoption. In terms of safety, the proportion of pharmacists’ prescription questions decreased significantly from 3.1% before to 0.3% after the initiative. Additionally, the proportion of prescription changes resulting from these questions decreased significantly, from 1.2% to 0%. The PBPM development process evaluated here could successfully form effective PBPMs, which have the potential to reduce physicians’ workload, indicating that the process detailed in this study could be applied to future protocol development.

## 1. Introduction

The problem of prolonged working hours among physicians in Japan has received significant attention recently, with numerous recommendations issued by the government. According to a report by the Ministry of Health, Labour and Welfare [1], physicians are the professionals most affected by long working hours, necessitating urgent intervention. Additionally, a rapid increase in the number of patients diagnosed with inflammatory bowel disease (IBD) has been observed in Japan [2]. Nationwide data indicate that the prevalence was 24 per 100,000 population in 1991, increasing to 76 per 100,000 during the period of 2003–2005 and reaching 228.5 per 100,000 in 2014 [3,4]. This corresponds to a rise in the estimated number of individuals with IBD from 29,700 in 1991 to 290,400 in 2014. In addition, the prevalence of ulcerative colitis (UC) in Japan has exhibited substantial growth, with reports showing an increase from 5 per 100,000 population in 2010 to 98 per 100,000 in 2019 [5]. Consequently, support from physicians involved in IBD care is anticipated to be critical for patients with IBD, further escalating the workload of these physicians.

Pharmacist-led prescription has been successfully implemented in several countries, including the USA, the UK, and Canada, to manage physicians’ tasks effectively. For example, collaborative drug therapy management (CDTM), a common practice in some countries, such as the USA, involves contractual partnerships between physicians and pharmacists to manage specific patient treatments, with pharmacists playing an active role in pharmacotherapy management [6]. This approach has been proven highly effective by numerous studies [7,8]. In Japan, the Japanese Society of Hospital Pharmacists advocates the use of protocol-based pharmacotherapy management (PBPM), which has also been recommended in documents related to the promotion of team-based healthcare by the Ministry of Health, Labour and Welfare [9]. PBPM enables the active involvement of pharmacists in roles that, like in many other countries, legally cannot include prescribing medications or ordering tests in place of physicians. Therefore, instead of directly adopting CDTM, Japan has chosen to implement PBPM, a solution that has demonstrated its effectiveness. PBPM simplifies interactions between physicians and pharmacists by establishing predetermined protocols that pharmacists can follow to process prescriptions. This management occurs within legal boundaries and follows the guidelines of the Ministry of Health, Labour and Welfare. The specifics of PBPM are delegated to individual healthcare facilities.

Naturally, provisional prescriptions drafted by pharmacists are legally recognized only once confirmed and approved by a physician. PBPM is an effective and robust management tool for shifting and sharing tasks from physicians to pharmacists. The Japanese Society of Hospital Pharmacists has published a guide, “Smooth Implementation and Practical Examples of Protocol Based Pharmacotherapy Management (PBPM) Ver. 1.0”, which serves as a reference for medical facilities across Japan to formulate their PBPMs [10]. Numerous studies have highlighted the practical applications and benefits of PBPM [11,12,13].

In the context of IBD care, PBPM plays a crucial role in managing the increasing complexity of treatment regimens. For example, PBPM facilitates the initiation and adjustment of therapies, such as corticosteroids and biologics, ensuring timely and consistent patient management. Pharmacists can use PBPM to address routine tasks, including monitoring therapeutic drug levels, managing medication adherence, and providing prophylactic measures to prevent infections associated with immunosuppressive treatments. By reducing the need for verbal instructions and streamlining workflows, PBPM improves communication efficiency and reduces the burden on physicians while maintaining high standards of patient safety.

Despite its establishment, in the field of IBD, PBPM has been reported to have a low adoption rate [14]. This suggests that even when protocols are in place, they are not always utilized effectively and thus do not contribute to the intended physician task shift/share. Although there are case collections and reports on PBPM initiatives in Japan [15,16], detailed reports on the adoption status or protocol development process are currently lacking.

This study aimed to retrospectively assess the effectiveness of the PBPM development process by evaluating the impact of a newly developed PBPM, which was determined by measuring how pharmacists were involved in the prescribing process.

## 2. Materials and Methods

### 2.1. Study Design

This study was a prescription-based retrospective study. The target prescriptions were judged using the electronic medical record’s checking system function according to the pre-determined PBPM criteria. These prescriptions were then analyzed for this study.

### 2.2. Additional Formulation of PBPM

Prior to this study, a total of 37 PBPMs had been established at our hospital. Among these, 10 protocols were designated for use in departments other than the IBD center, while 11 were specific to outpatient care. The remaining 16 protocols were common across all departments and applicable to inpatients at the IBD center. These 16 protocols served as the foundation for the development of additional IBD-specific PBPMs in this study (Table 1).

We reviewed records of provisional prescriptions made by pharmacists based on verbal instructions from physicians between October and December 2021. In this context, verbal instructions refer to specific directives from physicians regarding the treatment or medication for individual patients. For example, these instructions may include prescribing antibiotics to prevent infections associated with steroid therapy or continuing a patient’s current oral medications. Such instructions were communicated directly to pharmacists, who then created provisional prescriptions in accordance with the physician’s guidance. Two pharmacists from the IBD team selected records that contained universal and robust prescription instructions suitable for incorporation into the protocol. Sporadic instructions were excluded based on the expert judgment of two pharmacists from the IBD team. Ad hoc instructions referred to directives tailored to individual cases without general relevance, and patient-specific instructions, such as dose adjustments for unique physiological conditions, were also excluded. The selected records were classified using an open card sorting method, a qualitative technique where pharmacists grouped similar records into thematic categories without predefined criteria. The pharmacists aimed to group the records as broadly as possible while maintaining clinical relevance. Each group was assigned a descriptive label, and actionable protocols were formulated based on these labels. These protocols defined the specific actions for pharmacists in prescription management. The pharmacists and physicians of the IBD team reviewed the articulated protocols multiple times in January 2022 to refine the phrasing to ensure that the protocols accurately reflected the physicians’ intentions without any misinterpretation. The consultations also verified the feasibility of the protocol. After reaching a consensus, these protocols were formalized according to hospital regulations, resulting in an additional formulation of PBPM.

### 2.3. Participants

This study retrospectively investigated the involvement of pharmacists in providing prescriptions that were effective for inpatients treated at the IBD Center of Tsujinaka Hospital Kashiwanoha (hereafter referred to as “our hospital”) during the three months before and after the development of the new PBPM (October to December 2021 and February to April 2022). The evaluation examined whether the pharmacists were involved in issuing the prescriptions under review. Additionally, the prescription type, content, and patient demographics (sex, age, and IBD diagnosis) were analyzed. IBD diagnoses included UC, Crohn’s disease (CD), and other diseases managed at the IBD Center, including but not limited to Behcet’s disease. The number and details of errors related to pharmacist involvement in prescribing were also extracted.

### 2.4. Outcomes

The primary outcome measure of this study was the change in pharmacist involvement in prescription issuance before and after the implementation of the newly developed PBPM. Pharmacist involvement was categorized into three types: no pharmacist involvement (SP), provisional prescribing upon a physician’s directive (PPP), and prescribing based on PBPM protocols (PBPM-P). Detailed definitions of these categories are provided in the following section. This change was further examined according to prescription type. In the exploratory analysis, we performed factor analysis to identify the variables influencing pharmacist involvement in the issuance of provisional prescriptions. Additionally, a comparative analysis was conducted on the number of errors and prescription queries involving pharmacist participation.

### 2.5. Pharmacist Involvement in Prescription Issuance

In this study, we defined how pharmacists were involved in the prescription processes initiated by physicians. Specifically, pharmacist involvement was categorized into three types: (1) no pharmacist involvement (standard process: SP), where physicians managed all prescription tasks independently; (2) pharmacists creating a provisional prescription upon a physician’s explicit request or directive (pharmacist provisional prescribing: PPP); and (3) pharmacists autonomously drafting prescriptions based on established PBPM protocols (PBPM-P). Provisional prescriptions were created using the requesting physician’s function in the Fujitsu Electronic Medical Record System (HOPEEGMAIN GX). This was performed according to the hospital guidelines, confirming prescriptions issued based on physician requests and those based on PBPM.

### 2.6. Types and Content of Prescriptions

The categories of prescriptions at our hospital follow the typical classification in Japan, divided into two types: those for oral and topical medications (hereafter referred to as “prescriptions”) and those for injectable medications (hereafter referred to as “injection prescriptions”). Analyses were conducted for each type, as required.

### 2.7. Safety of Prescription Practices

Safety in this study was evaluated through two distinct measures. First, we examined the number of prescription questions raised by pharmacists during their audits. These questions were triggered when pharmacists identified potential issues warranting clarification or modification, including concerns about dosage and administration schedules, the appropriateness of the medication’s indication, potential allergies or contraindications, risks of side effects or adverse drug reactions, possible drug–drug interactions, and verification grounded in clinical and pharmaceutical knowledge. The total number of questions and subsequent modifications to prescriptions were recorded, and the ratios of these occurrences to the total number of prescriptions were calculated and compared between the periods before and after the initiative. Second, the safety of the prescription process was assessed by tracking all errors that occurred during pharmacist involvement in the provisional prescription processes under PPP and PBPM-P. Errors were identified during the pharmacist’s provisional prescription entry, as well as at all stages of these processes. All identified errors were aggregated and analyzed as proportions of the total number of prescriptions within the PPP and PBPM-P categories. These proportions were compared between the pre- and post-initiative periods to evaluate the impact of the initiative on prescription safety.

### 2.8. Statistical Analyses

Statistical analyses were conducted using chi-square tests and logistic regression. The chi-square test was used to examine any significant changes in the ratio of pharmacist involvement in prescription issuance before and after the intervention. If significant, Pearson’s chi-square residuals were calculated, as required, for a more detailed analysis. Additionally, factors affecting pharmacist involvement in prescribing were explored using multiple logistic regression. The analysis involved several steps utilizing a multiple logistic regression model. Initially, pharmacist involvement in prescription issuance was treated as the outcome, with SP and PPP as the reference category, and univariate analyses were conducted for provisional prescriptions based on PBPM-P. Factors showing a *p*-value of <0.10 in the univariate analyses were forcibly entered as explanatory variables in the multiple logistic regression. The presence of multicollinearity among independent variables was assessed using the variance inflation factor (VIF), with values above 10 indicating multicollinearity and thus excluded from the model. The goodness of fit of the model was post-estimated using the C Index and considered well-fitted if it was greater than 0.70 and poorly fitted if the lower limit of the 95% confidence interval (CI) was below 0.50. The results are presented as crude odds ratios (ORs) or adjusted odds ratios (adj-ORs), with the significance level set at 0.05 for all analyses. Statistical analyses were performed using R (The R Foundation for Statistical Computing, Vienna, Austria, version 4.1.1).

## 3. Results

### 3.1. Prescriptions Analyzed

A total of 1259 prescriptions were analyzed in this study. Before the initiative, there were 586 prescriptions (oral/topical, 128; injection, 458); after the initiative, there were 673 prescriptions (oral/topical, 242; injection, 431). The median age of the patients for whom prescriptions were issued was 42 years before the initiative and 36 years after the initiative. The breakdown of IBD conditions was as follows: UC accounted for 271 cases before and 497 cases after the initiative, whereas CD accounted for 286 cases before and 114 cases after the initiative (Table 2).

### 3.2. Implementation of New PBPM Protocols

**Figure 1:**
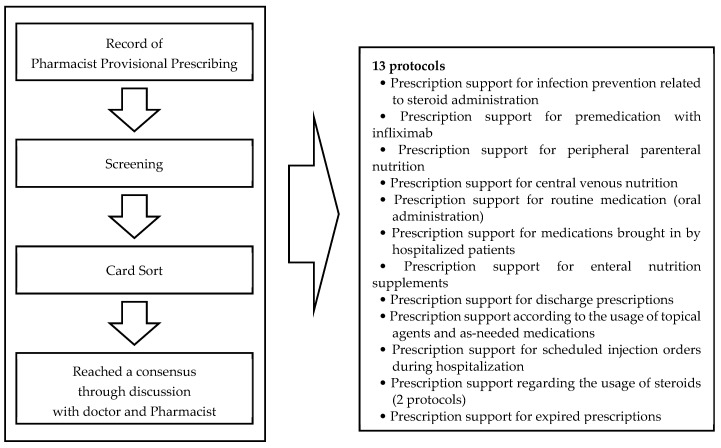
Development and implementation process of additional PBPMs. The protocols were generated using several processes. We developed 13 protocols for these 12 items. Each protocol specifies the actions that pharmacists should take regarding prescriptions.

Following a predetermined method, the records were classified into 12 categories, from which 13 protocols were developed. These protocols were discussed and adjusted in consultation with IBD physicians, resulting in a final addition of 13 new PBPMs (Figure 1). The additional protocols were specific to patients with IBD. For example, for patients receiving more than 30 mg/day of steroids in prednisone equivalents, pharmacists were authorized to initiate the process of provisional prescription of antibiotics and gargle solution for infection prevention after verifying the patient’s allergy history and confirming its applicability. This meant that the study compared two conditions: before the initiative, when 16 PBPMs were available, and after the initiative, when 13 additional PBPMs were added, resulting in a total of 29 PBPMs.

### 3.3. PBPM Application Rates

When comparing the periods before and after the initiative, the overall prescription data showed significant changes. Initially, the breakdowns were 400, 180, and 6 for SP, PPP, and PBPM-P, respectively; however, these changed to 325, 179, and 169, respectively, post-initiative. Chi-square tests indicated a significant difference in pharmacist involvement before and after the initiative (X^2^ = 154.3, df = 2, *p* < 0.001, Cramer’s V = 0.35). Further analysis using adjusted standardized residuals (adj-R) showed values exceeding 1.96 for both SP and PBPM-P, suggesting a significant decrease in SP and a significant increase in PBPM-P after the initiative. When analyzed by prescription type, the oral/topical prescriptions shifted from 36 SP, 86 PPP, and 6 PBPM-P pre-initiative to 52 SP, 130 PPP, and 60 PBPM-P post-initiative, showing a significant difference (X^2^ = 23.1, df = 2, *p* < 0.001, Cramer’s V = 0.24). Residual analysis indicated a significant increase only for PBPM-P. The pre-initiative figures for injectable prescriptions were 364 SP, 94 PPP, and 0 PBPM-P, which changed to 273 SP, 49 PPP, and 109 PBPM-P post-initiative. This also showed a significant difference (X^2^ = 135.4, df = 2, *p* < 0.001, Cramer’s V = 0.39), with residual analysis showing significant decreases in SP and PPP, while PBPM-P significantly increased (Table 3).

### 3.4. Factor Analysis of PBPM Implementation

This analysis utilized logistic regression to examine the factors influencing the adoption of PBPM-P compared with SP and PPP, categorizing SP and PPP as the reference groups and PBPM-P as the target group. The results of the univariate analysis are presented in Table 4. As pre-specified, factors with an OR below 0.10 in the univariate analysis were included in the multivariate analysis to ensure comprehensive consideration of potentially influential variables. The effort variable, reflecting changes before and after the initiative, showed a substantial increase in the likelihood of achieving the desired outcome, maintaining its significance even in multivariate analysis with an OR of 39.47 (95% CI: 16.87–92.33, *p* < 0.001). The other factors examined included sex, age, disease state, and prescription type. Sex was not significantly associated with the outcome, whereas age was inversely associated with positive outcomes, decreasing slightly with each additional year (OR = 0.98, 95% CI: 0.9–70.99, *p* < 0.001). Patients with UC had significantly lower odds of achieving a positive outcome than those with CD (OR = 0.16, 95% CI: 0.05–0.52, *p* = 0.002), while other disease categories did not show significant differences. Injectable versus oral prescription types showed lower odds for injectables (OR = 0.64, 95% CI: 0.46–0.90, *p* = 0.01), although this factor was not significant in the multivariate context (Table 3). The Hosmer–Lemeshow test confirmed the adequacy of the model (*p* = 0.138). It demonstrated good discriminatory ability, with an area under the curve (AUC) of 0.83 (95% CI: 0.80–0.83).

### 3.5. Safety Outcomes of the Initiative

**Figure 2:**
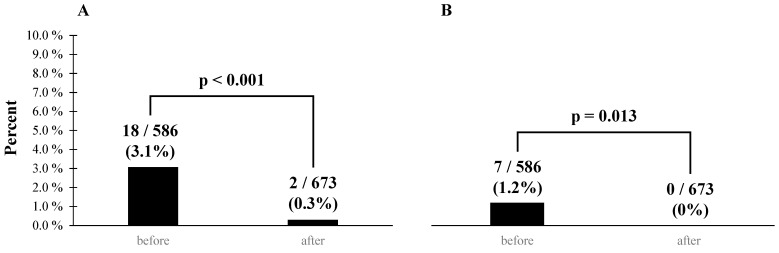
Comparison of pharmacist prescription questions and errors pre- and post-initiative. (**A**) Number of pharmacist prescription questions before and after initiation. (**B**) Number of prescription changes resulting from the pharmacists’ questions before and after the initiative.

Before the initiative, there were 18 instances of prescription questions by pharmacists (3.1%, 18/586), which decreased to 2 instances post-initiative (0.3%, 2/673), showing a significant reduction (*p* < 0.001). Additionally, of the pre-initiative prescription questions, seven (1.2%, 7/586) resulted in changes to prescriptions, whereas no changes occurred post-initiative (0%, 0/673), also showing a significant decrease (*p* = 0.013) (Figure 2). Errors in pharmacists’ prescription entries, including those involving PBPM-P, did not exist before the initiative (0%, 0/186), and there were two instances (0.6%, 2/348) after the initiative; however, this difference was not statistically significant (*p* = 0.770). These errors included one electronic medical record registration error and one omission in prescription transcription. Both errors were promptly corrected, and no patient was harmed.

## 4. Discussion

This study utilized insights from past pharmacist involvement in prescription processes to successfully develop and expand the application of PBPMs. Although there are numerous reports on the effectiveness of PBPM in Japan, to the best of our knowledge, no study has elaborated on the process of formulating PBPMs. Following the initiative described in the present study, there was a significant and consistent increase in the use of PBPM-P across all prescription categories. Even with adjustments, the current initiative has proven to be a significant factor in promoting PBPM. These findings suggest that this initiative is beneficial in creating effective PBPMs.

This study has several notable strengths. First, it provides a detailed and systematic approach to the development of PBPM, tailored specifically to IBD care. By analyzing real-world data from past pharmacist-led provisional prescriptions, the study bridges the gap between theoretical protocol design and practical application in clinical settings. Second, the methodology, which involved iterative discussions and collaboration between pharmacists and physicians, highlights an innovative and replicable approach to protocol development. Finally, the findings underscore the potential of PBPM to significantly reduce physician workload and improve the efficiency of multidisciplinary teamwork in healthcare settings.

This study also highlights the value of analyzing past practices. Focusing on a specific medical department simplifies the selection of experts for protocol creation. If the tasks within a department are somewhat standardized, they can be effectively translated into protocols, naturally leading to the development of more applicable protocols. Previous surveys have established that pharmacist involvement in rounds and participation in clinical conferences increase the delegation of provisional prescription upon a physician’s request or directive. However, the application rate of PBPM remained low [14]. This could indicate that pharmacists become more proactive in prescription activities when they are allowed to alter or innovate their involvement in routine clinical practice. However, this does not necessarily extend to the applications of PBPM.

Thus, the development of PBPM is crucial for making it more practical and widely utilized. However, pharmacists in Japan cannot independently prescribe medications currently. The distinction between PPP and PBPM-P in this study lies in the source of directive initiation. While PPP involves a physician directing a pharmacist, leading to pharmacist involvement in prescription issuance, PBPM-P enables pharmacists to autonomously engage in prescription issuance based on established protocols. The subsequent process after a pharmacist issues a provisional prescription remains the same in both models. However, if physicians and pharmacists follow appropriate methodologies, pharmacists can be involved in the prescription process. The formation of provisional prescriptions by pharmacists can facilitate physicians’ tasks even when physicians are not in the electronic medical record system. Reports suggest that pharmacists’ engagement in prescribing activities has been beneficial [17,18], highlighting the potential for pharmacists to significantly assist in managing physician tasks. While the creation of provisional prescriptions by pharmacists was active in this study and prior research, it necessitated verbal directives, implying high communication density due to the frequency of such interactions. In contrast, PBPM-P operates under predefined protocols, thus reducing the need for verbal communication and lowering communication density.

Tahara and colleagues have reported a negative correlation between the amount of communication and teamwork [19], indicating that PBPM could clarify actions and improve teamwork. This also implies that PBPM reduces communication costs between doctors and pharmacists, potentially easing the burden on physicians. Therefore, to assist physicians effectively, pharmacists must do more than merely facilitate provisional prescriptions. To meaningfully reduce physician workload, the application rate of PBPM must be enhanced. By streamlining communication and clarifying prescription procedures through PBPM, the healthcare system can improve efficiency and potentially enhance patient care outcomes by ensuring that pharmacists are better integrated into the care delivery process, without the constant need for direct physician oversight.

Risk assessment in tasks based on PBPM is crucial. Although creating provisional prescriptions by pharmacists may carry risks, as they are not directly legislated, this study did not observe increased risks associated with this initiative. Intriguingly, the initiative reduced the number of prescription questions and subsequent changes, which often indicate prescription errors. This suggests that the active implementation of PBPM helps suppress prescription errors. Furthermore, reducing errors aligns with broader goals of enhancing patient safety and care quality. Poh et al. reported that when pharmacists prescribe drugs according to protocols, they adhere to the dosing guidelines and significantly reduce prescription errors [20]. Moreover, Lloyd et al. reported that pharmacists who received training in discharge prescription transcription had a lower error rate than doctors during discharge [21]. These reports support the findings of our study. In other words, the implementation of PBPM not only serves to reduce the workload of physicians but also provides a beneficial approach that contributes to patient safety. Because the analyses in this study were exploratory, further research focusing on operational errors within PBPM may be necessary.

This study has some limitations. First, the legal framework governing prescriptions varies significantly across countries and is unique even in Japan. This study was conducted at a single facility, which may limit the generalizability of the findings. However, the results indicate that pharmacists can deepen their involvement in prescription issuance by analyzing past cases, which is likely applicable in settings where legal restrictions on pharmacists are similar to those in Japan. Second, the process of formulating additional PBPMs in this study involved the card sorting method, which is an inherently narrative approach and may lack the systematic rigor that methods like text mining or clustering can provide. Although there is room for improvement in the development of a more theoretical approach, incorporating a narrative method by experts into protocol articulation is considered effective, as supported by the findings of this study. Third, this study focused primarily on applying PBPM and its impact on physician task shifting/sharing without clarifying its effects on patient outcomes. Although the beneficial effects of PBPM on patient outcomes have been extensively reported in Japan, these effects were not verified in the present study. However, the reduction in prescription queries and changes owing to the initiative can indirectly contribute to patient safety. This remains an area for future research. Finally, provisional prescriptions based on PBPM may not sufficiently assist in reducing physician tasks because they require physician approval to become formal prescriptions. In other countries, pharmacist-led models, such as the CDTM, have proven beneficial [22], highlighting the potential advantages of granting more autonomy to pharmacists [23]. This suggests a possible direction for future policy and practice changes in Japan, aiming to enhance the effectiveness of pharmacist involvement in patient care.

Therefore, a system similar to the CDTM that grants prescription autonomy to pharmacists is also desirable in Japan. The implementation of such a system may involve significant challenges, including legislative changes. Thus, further research on PBPM is essential to verify its effectiveness and safety. This could pave the way for the establishment of frameworks enabling pharmacists to play a more active and autonomous role in patient care management.

## 5. Conclusions

This study underscores the essential contribution of analyzing pharmacists’ provisional prescriptions in developing effective PBPM systems. Key variables analyzed in this study included the rates of SP, PPP, and PBPM-P. These variables provided valuable insights into the changes in prescription processes before and after the initiative.

The implementation of new PBPM protocols significantly increased the use of PBPM-P while reducing SP and PPP rates. This demonstrates their effectiveness in optimizing prescription processes, enhancing communication, and improving task distribution between pharmacists and physicians. Additionally, the observed reduction in prescription questions and changes highlights the initiative’s impact on improving safety and efficiency.

Looking ahead, the findings of this study provide a solid foundation for exploring the broader application of PBPM in diverse medical fields. Expanding the use of PBPM has the potential to reduce physician workload, enhance interdisciplinary collaboration, and improve patient safety. Future research should evaluate the applicability of PBPM in various clinical settings, focusing on long-term patient outcomes, cost effectiveness, and interdisciplinary workflow optimization. These efforts will help establish PBPM as a cornerstone for improving healthcare efficiency and outcomes, paving the way for its integration into broader medical practices.

## Figures and Tables

**Table 1:** Overview of PBPMs already implemented before the initiative.

Classification	No. of Prepared Protocols	Description
Omission of prescription question	3	This protocol stipulates the omission of some steps in the usual process of prescription verification and inquiry. It includes procedures to simplify or omit inquiries for clear prescriptions or routine cases.
Prescription support	7	This protocol includes a system where pharmacists support the prescription process by adjusting dosages or modifying prescriptions when necessary due to patient background or drug interactions.
Prescription completion	5	This protocol includes procedures to support pharmacists in completing the process for prescriptions that are incomplete or partially missing.
Dispensing support	1	This protocol includes the process wherein pharmacists adjust dispensing instructions given by doctors based on the patient’s condition and other factors.

The protocols listed in the table were established before the initiative. Of the 37 PBPMs that had already been established, the table displays 16 that were applicable to the subjects of this study.

**Table 2:** Characteristics of studied prescriptions.

	Overall	Initiative ^a^	
		Before	After
Number of prescriptions	1259	586	673
-Oral and topical medication	370	128	242
-Injectable medication	889	458	431
Sex			
-Male	953	422	531
-Female	306	164	142
Age (IQR)	39 (28–49)	42 (30–52)	36 (21–43)
Disease			
-CD	400	286	114
-UC	768	271	497
-Others ^b^	91	29	62

^a^ This refers to a series of interventions through which the PBPM was developed. ^b^ Other diseases managed at the IBD Center besides UC and CD, including but not limited to Behcet’s disease. IQR, inter-quartile range; CD, Crohn’s disease; UC, ulcerative colitis.

**Table 3:** Changes in pharmacist involvement in prescriptions before and after the initiative.

Label		Overall		Medication			
				Oral and Topical		Injectable	
		Initiative		Initiative		Initiative	
		Before	After	Before	After	Before	After
SP	Observed	400	325	36	52	364	273
	Expected	337.5	387.5	30.4	57.6	328.2	308.8
	Std_Residuals	3.41	−3.18	1.01	−0.73	1.98	−2.04
	Adj_Std_Residuals	7.15	−7.15	1.43	−1.43	5.33	−5.33
PPP	Observed	180	179	86	130	94	49
	Expected	167.1	191.9	74.7	141.3	73.7	69.3
	Std_Residuals	1.00	−0.93	1.30	−0.95	2.37	−2.44
	Adj_Std_Residuals	1.61	−1.61	2.50	−2.50	3.71	−3.71
PBPM-P	Observed	6	169	6	60	0	109
	Expected	81.5	93.5	22.8	43.2	56.2	52.8
	Std_Residuals	−8.36	7.80	−3.52	2.56	−7.49	7.72
	Adj_Std_Residuals	−12.32	12.32	−4.81	4.81	−11.49	11.49

SP, standard process; PPP, pharmacists’ provisional prescription; PBPM-P, process through which pharmacists create provisional prescriptions based on PBPM.

**Table 4:** Logistic regression analysis of factors influencing PBPM implementation.

Factor	Crude OR (95% CI)	*p*-Value	adj. OR (95% CI)	*p*-Value
Initiative				
-Before (ref)	-	-	-	-
-After	32.41 (14.23–73.81)	<0.001	39.47 (16.87–92.33)	<0.001
Sex				
-Male (ref)	-	-		
-Female	0.72 (0.48–1.07)	0.106		
Age (per 1-year increase)	0.97 (0.95–0.98)	<0.001	0.98 (0.97–0.99)	<0.001
Disease				
-CD (ref)	-	-	-	-
-UC	0.18 (0.06–0.57)	0.004	0.16 (0.05–0.52)	0.002
-Others	1.37 (0.96–1.95)	0.086	0.50 (0.33–0.76)	0.001
Prescription category				
-Oral and topical medication (ref)	-	-	-	-
-Injectable medication	0.64 (0.46–0.90)	0.009	0.82 (0.57–1.18)	0.275

The left side of the table shows the results of the univariate analysis, while the right side shows the results of the multivariate analysis. SP and PPP are used as reference groups, with PBPM as the target group. Other diseases managed at the IBD Center besides UC and CD, including but not limited to Behcet’s disease. OR, odds ratio; adj. OR, adjusted odds ratio; CI, confidence interval; ref, reference; CD, Crohn’s Disease; UC, ulcerative colitis.

## Data Availability

The datasets generated during and/or analyzed during the current study are available from the corresponding author upon reasonable request.
